# Decomposition of air conditioning electricity consumption based on effective duration

**DOI:** 10.1371/journal.pone.0308459

**Published:** 2024-08-08

**Authors:** Chao Xun, Huan Zheng, Zhaoyu Han

**Affiliations:** 1 State Grid Fujian Electric Power Co., Ltd., Fuzhou, China; 2 Economic and Technical Research Institute, State Grid Fujian Electric Power Co., Ltd., Fuzhou, China; 3 Department of Automation, North China Electric Power University, Baoding, China; Aalto University, FINLAND

## Abstract

As the share of air conditioning electricity consumption within total grid electricity consumption grows, the decomposition of such consumption becomes increasingly crucial for assessing electricity usage patterns, devising consumption scheduling strategies, and maintaining the stability of the power grid. Although there is a strong correlation between apparent temperature and air conditioning electricity consumption, the literature currently available seldom explores the impact of apparent temperature on this consumption. Moreover, there is a scarcity of effective assessment indices to evaluate the efficacy of air conditioning electricity consumption breakdown. This study introduces a method for decomposing electricity consumption from air conditioning units, utilizing effective duration as a basis to tackle these issues. By employing an apparent temperature model as a constraint, this approach identifies the effective operating time of air conditioning and constructs a constrained convex optimization problem to estimate air conditioning power usage. Additionally, a novel evaluation index for the effectiveness of air conditioning electricity consumption decomposition is proposed, which includes penalties for negative decomposed consumption, alongside the traditional consistency index. Comparative experiments are conducted using real electricity consumption data from Fujian Province. Empirical results indicate that the methodology for air conditioning electricity consumption decomposition presented in this paper aligns more closely with actual conditions. Furthermore, the evaluation metrics introduced for the decomposition of air conditioning electricity consumption are adept at precisely gauging the quality of the air conditioning electricity consumption data.

## Introduction

Air conditioning electricity consumption decomposition refers to the separation of the electricity consumed by air conditioning equipment from the total grid electricity consumption. As of the end of 2022, the National Bureau of Statistics of China reported 133.9 air conditioners per 100 households in China, reflecting the increasing prevalence of air conditioning. Singh J et al. [1] conducted a survey on Indian household electricity usage, revealing a high penetration rate of air conditioners in urban Indian households. The survey also demonstrated that the utilization of air conditioners and the number of units per household significantly affect the total electricity consumption. Randazzo T et al. [2] investigated the relationship between air conditioning and electricity consumption in eight temperate industrialized countries, finding that households with air conditioners, influenced by climate change, exhibit higher electricity consumption. Consequently, the average annual electricity costs for households equipped with air conditioners are 35% to 42% greater than those without air conditioning. It is anticipated that, with societal development, the power consumed for air conditioning will significantly impact overall electricity consumption. Therefore, it is of considerable significance to examine the characteristics of air conditioning electricity consumption and to accurately quantify its magnitude. This analysis can assist utilities in forecasting electricity sales and grid loads, facilitating timely grid scheduling to ensure stable operation.

Aqilah N et al. [3] conducted a survey on the air conditioning electricity consumption of Malaysian residences, utilizing sensors deployed within homes to gather the data. Yuan Y et al. [4] investigated and analyzed the air conditioning electricity consumption of offices in 21 universities, also employing sensor deployment to obtain their data. Chou J S et al. [5] successfully predicted office air conditioning electricity consumption, with their historical data similarly collected via sensor deployment. Although sensor deployment can yield precise air conditioning electricity consumption data, it is currently infeasible to implement real-time monitoring and statistical analysis of air conditioning electricity consumption across diverse industries and on a large scale due to practical constraints. Consequently, decomposing electricity usage using total grid power consumption data remains the most efficient method for acquiring air conditioning electricity consumption data.

Air conditioning electricity consumption is fundamentally the cumulative air conditioning load over time. Consequently, the same techniques employed to decompose air conditioning loads can also be utilized to decompose air conditioning electricity consumption. Common methods for decomposing air conditioning load encompass the peak-load comparison method (PLCM) [6–8], the basic-load comparison method (BLCM) [9–11], the maximum temperature difference analysis (MTDA) [12], and various other approaches [13].

Although PLCM, BLCM, and MTDA all partition the overall grid load into base load and air conditioning load, their underlying theories differ. PLCM calculates the maximum air conditioning load by comparing the maximum load in the third quarter with the maximum load in months without an air conditioning load, focusing on the overall load change patterns of the power grid. Shi Feng et al. [7] applied this technique to analyze Chongqing’s summer air conditioning load and proposed strategies to manage the increase in peak-period air conditioning load and improve grid load characteristics. While PLCM involves straightforward calculations, it necessitates data for the entire year, resulting in a time lag. Additionally, the method’s precision is compromised and susceptible to the influence of anomalies. Rong Xiuting et al. [8] integrated the temperature gradient method with the maximum load comparison method to mitigate the issue of insufficient sample space. BLCM, on the other hand, calculates the load difference between the load curve and the curve representing the typical load during various seasons, treating this difference as the air conditioning load. Liao Feng et al. [9] utilized BLCM to analyze past grid loads in Changde and explored the relationships between different load components and influencing factors. Xiong Longzhu et al. [10] investigated the impact of temperature on air conditioning load in Zhangjiajie, while Shi Jing et al. [11] examined the relationship between summer air conditioning load and temperature, as well as the variables affecting air conditioning electricity usage. MTDA [12] treats the difference between the two-day loads on days with sudden temperature fluctuations as the direct air conditioning load.

Despite their straightforward calculating flow, the aforementioned approaches have certain shortcomings:1) Air conditioning load calculations lag: PLCM and BLCM employ load data from spring and autumn as the foundation for no-air conditioning load data, resulting in a certain lag in air conditioning load calculations. 2) Disregarding the natural increase of the grid’s base load: These solutions overlook the normal increase of the grid’s base load over time and instead use the maximum load of a given day or the mean of maximum loads as the base load. 3) Subjective selection of typical months or days: The selection of typical months or days in these methods is subjective, compromising the reliability of the results due to the influence of outliers.

Other methods also consider the overall grid load as the sum of the base load and air conditioning load, and then employ different approaches from the aforementioned methods to obtain the base load, subsequently decomposing the air conditioning load. For instance, Wang Ruimiao et al. [13] used the correlation coefficient between temperature and load to select data for typical spring and autumn days and applied filtered load data to fit a base load curve. Based on this curve, the air conditioning load was decomposed. However, the usefulness of this method is limited as the correlation coefficient can only depict a linear relationship between temperature and load.

Apart from the aforementioned methods, other researchers have explored different approaches to obtain air conditioning electricity consumption data. Chen et al. [14] conducted a quantitative analysis of the relationship between residential air conditioning power and indoor/outdoor temperatures, as well as the number of air conditioners. They simplified the air conditioning power calculation model by leveraging commonalities among residences, leading to the development of an aggregated calculation model for residential air conditioning. This model allows for the calculation of air conditioning power for individual residences. Huang et al. [15] predicted the distribution of indoor thermal environment based on the Block-Gebhart (B-G) model and utilized radiant and convective heat exchange load calculation methods to determine stratified air conditioning loads. However, this method is only applicable to large-scale buildings with low-side wall air supply systems. Manivannan et al. [16] employed machine learning techniques to separate air conditioning loads from the total residential load by analyzing characteristic information, such as voltage, of different household appliances. However, this approach requires high-quality data and primarily focuses on calculating the air conditioning load for residential buildings. While these methods have achieved a certain level of accuracy, and in some cases high precision, in calculating air conditioning loads, they are limited to specific types of buildings or locations, with relatively narrow application scenarios. As a result, they are unable to adequately decompose air conditioning electricity consumption across a wide range of diverse industry scenarios.

Studies [8–11] indicate that air conditioning electricity consumption is closely related to ambient temperature. However, it depends not only on temperature but also on apparent temperature—the perceived temperature experienced by individuals. Furthermore, user behavior, such as turning on the air conditioner after a certain threshold of perceived discomfort, significantly contributes to air conditioning electricity usage. Current methods for decomposing air conditioning load often fail to account for user-perceived temperature. Additionally, these methods frequently produce negative values for air conditioning load decomposition, and the evaluation indices for these methods do not penalize negative values, resulting in less objective assessments.

To address these issues, this paper proposes a novel method for decomposing air conditioning electricity consumption and introduces a consistency index for this purpose. The main contributions are as follows:

- A decomposition method for air conditioning electricity consumption is developed based on effective duration. This approach calculates the effective operation time of air conditioning using an apparent temperature model and employs this as a constraint in a constrained convex optimization problem to estimate the electricity consumed for cooling. The convex optimization model is then solved to decompose air conditioning electricity consumption.
- The consideration of negative electricity consumption scores to evaluate the effectiveness of air conditioning electricity consumption decomposition. A new assessment index for air conditioning electricity usage decomposition is introduced by adding a penalty for negative electricity consumption to the traditional consistency index.
- The proposed decomposition method and its evaluation index are validated through comparative experiments using real electricity consumption data from Fujian Province.

## Methodology

The main topics covered in this section are the linear decomposition method for air conditioning electricity consumption, the BLCM method, and the structural features of electricity consumption data.

### Typical days and structural characteristics of electricity consumption data

**Fig 1:**
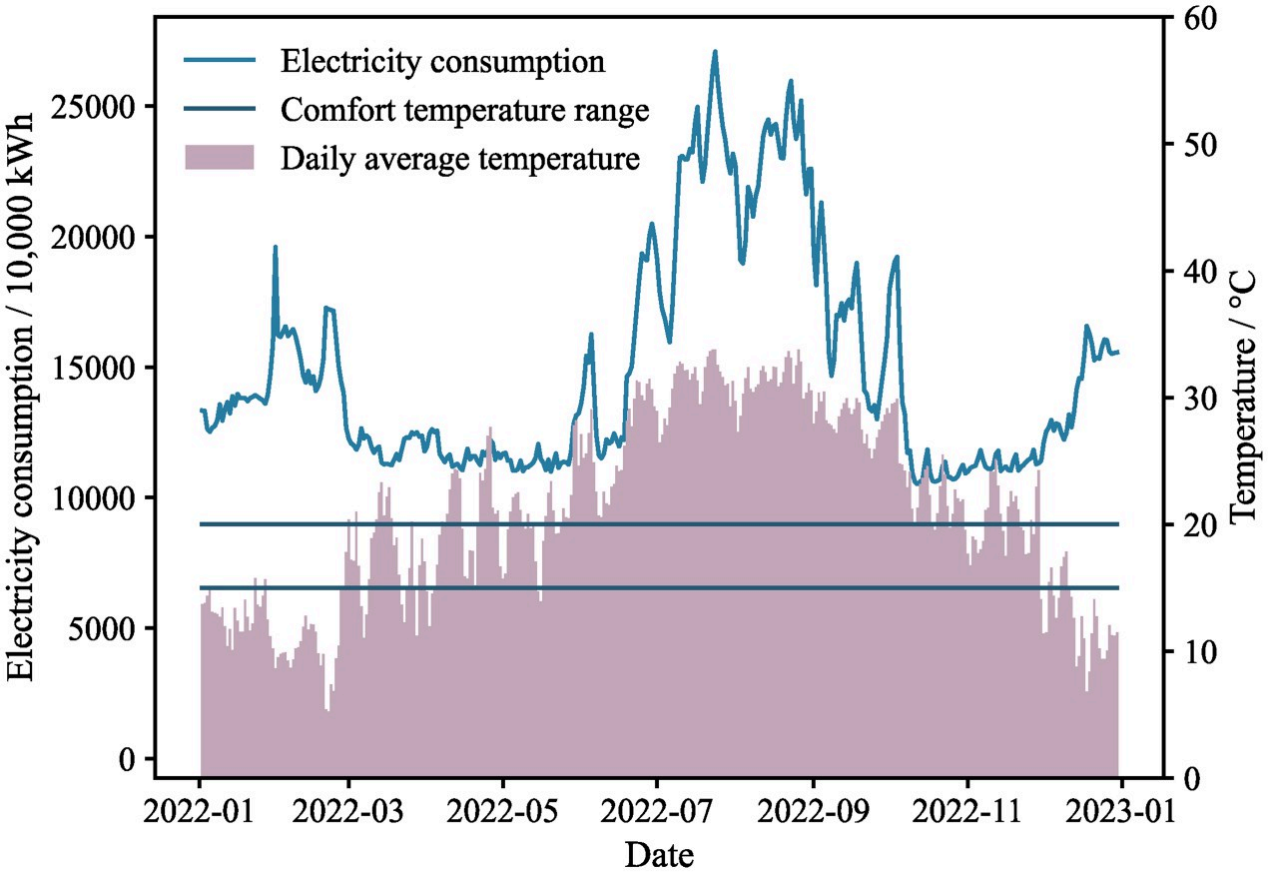
Electricity consumption for urban and rural residents in 2022.

Fig 1 presents the electricity consumption for urban and rural residents in Fujian province in 2022 alongside daily average temperatures.

It is evident that the peak electricity consumption among both urban and rural residents occurs during the summer months of July, August, and September. This surge is attributable to the widespread use of air conditioning systems, necessitated by the consistently high temperatures, with daily maxima exceeding 25°C. Conversely, the lowest electricity usage is observed in March, April, May, as well as November, aligning with the milder temperatures of spring and autumn, which typically fall within the comfort zone, thus obviating the need for air conditioning. In summary, there is a robust correlation between the summertime peak in electricity consumption and the operation of air conditioning systems. Additionally, significant increases in electricity usage are noted in February and October, coinciding with the Spring Festival and National Day holidays, respectively, when air conditioner usage and home occupancy are heightened. To mitigate the confounding effects of workdays and holidays, the data has been segregated into two distinct subsets: holidays and workdays.

In this study, the total grid electricity consumption is categorized into two components [13, 17, 18]: air conditioning electricity consumption and baseline electricity consumption, excluding air conditioning electricity consumption (hereafter referred to as baseline electricity consumption). The total grid electricity consumption encompasses the aggregate electricity usage by all grid users within a specified timeframe. Air conditioning electricity consumption denotes the electricity consumed due to the operation of air conditioning systems by users, which is influenced by temperature and other environmental factors [11–13]. Baseline electricity consumption represents the residual electricity usage in production and daily life after deducting air conditioning electricity consumption. This component is influenced by economic scale complexity, population, and other socio-economic factors [19], exhibiting varying patterns of change across different industries and years.

Building on the structural properties of electricity data, the concepts of typical and atypical days are introduced. A typical day is characterized by total grid electricity consumption that includes only baseline electricity consumption, with no air conditioning electricity consumption. Such days frequently occur during spring and autumn when ambient temperatures are comfortable and air conditioning is not necessary. Conversely, atypical days involve both baseline and air conditioning electricity consumption within the total grid electricity consumption on a given day. These days are more prevalent during periods of uncomfortable temperatures necessitating air conditioning, such as during hot summer days.

In summary, the structure of electricity consumption data can be represented as:

$$\begin{array}{c} \left\{ \begin{array}{cc} & E_{i}=C_{i},i\in \phi_{0}\\ & E_{i}=C_{i}+A_{i},i\in \phi \backslash \phi_{0} \end{array}\right. \end{array}$$ (1)

where,*E*_*i*_, *C*_*i*_ and *A*_*i*_ are the total grid electricity consumption, baseline electricity consumption, and air conditioning electricity consumption for day *i*, respectively; *ϕ* is the total dataset; *ϕ*_0_ is the dataset containing typical days; *ϕ*∖*ϕ*_0_ is the dataset containing all other days excluding typical days.

### BLCM

The BLCM is a straightforward and user-friendly approach for estimating air conditioning loads, particularly suitable when high precision is not a critical requirement [9–11]. Given that electricity consumption represents the aggregate load on the power grid over time, the BLCM can also be employed to calculate air conditioning electricity consumption.

The BLCM methodology designates spring and autumn days as typical days. The fundamental principle involves establishing the baseline electricity consumption for the summer months by averaging the daily total grid electricity usage observed on typical days during the spring and fall seasons. The steps to calculate summer air conditioning electricity consumption using the BLCM are as follows: 1) Identify spring and autumn days as typical days. 2) Calculate the summertime baseline electricity consumption by averaging the daily total grid electricity consumption on these typical days. 3) Determine the air conditioning electricity consumption by subtracting the baseline power usage from the daily total grid electricity consumption during the summer months. For winter baseline electricity demand calculations, the BLCM utilizes autumn and subsequent spring days as typical days.

The BLCM incorporates the natural growth of baseline electricity consumption from a mathematical statistical viewpoint. However, it assumes a linear increase in baseline power consumption, which may not accurately reflect the complex, non-linear variations in baseline electricity consumption across different sectors. Consequently, the method’s applicability is somewhat constrained.

### Linear decomposition method for air conditioning electricity consumption

Although the BLCM accounts for the natural increase in baseline electricity consumption, its strategy of employing the average total electricity consumption from spring and autumn as the summer baseline may reduce the accuracy of the decomposed air conditioning electricity consumption. To circumvent this limitation, the linear decomposition method for air conditioning electricity consumption, also referred to as the linear decomposition method, utilizes a simple linear model to estimate the baseline electricity consumption during summer, on the assumption that the baseline’s natural fluctuation follows a linear pattern. This method can be represented as follows:

$$\begin{array}{c} \left\{ \begin{array}{cc} & k=\frac{E_{i+1}-E_{i}}{t_{i+1}-t_{i}},\;i\in \phi \\ & C_{i}=kt_{i}+b,\;i\in \phi \backslash \phi_{0} \end{array}\right. \end{array}$$ (2)

where, *E*_*i*_ and *E*_*i*+1_ are the average daily total grid electricity consumption for month *i* and month *i* + 1, respectively, both of which are selected from the dataset of typical days; *t*_*i*_ is a time variable with month as the unit; *b* is a constant.

The linear decomposition method adeptly captures the linear growth of baseline electricity consumption. However, its monthly calculation approach results in the generation of only one baseline electricity consumption value per month, which limits the method’s precision in decomposing air conditioning electricity consumption. This limitation constrains the accuracy of the air conditioning electricity consumption estimates derived from this method. In real-world applications, months following typical days often yield negative air conditioning power usage statistics, underscoring the need for methodological improvements.

## Air conditioning electricity consumption decomposition method based on effective duration

This section presents the apparent temperature model, the decomposition approach based on effective duration, the constrained decomposition method for air conditioning power use, and the computation of effective duration. Building on the restricted decomposition approach for air conditioning electricity consumption, the latter method takes into account the impact of apparent temperature-based air conditioning effective duration on the air conditioning electricity consumption.

### Constrained decomposition method for air conditioning electricity consumption

**Fig 2:**
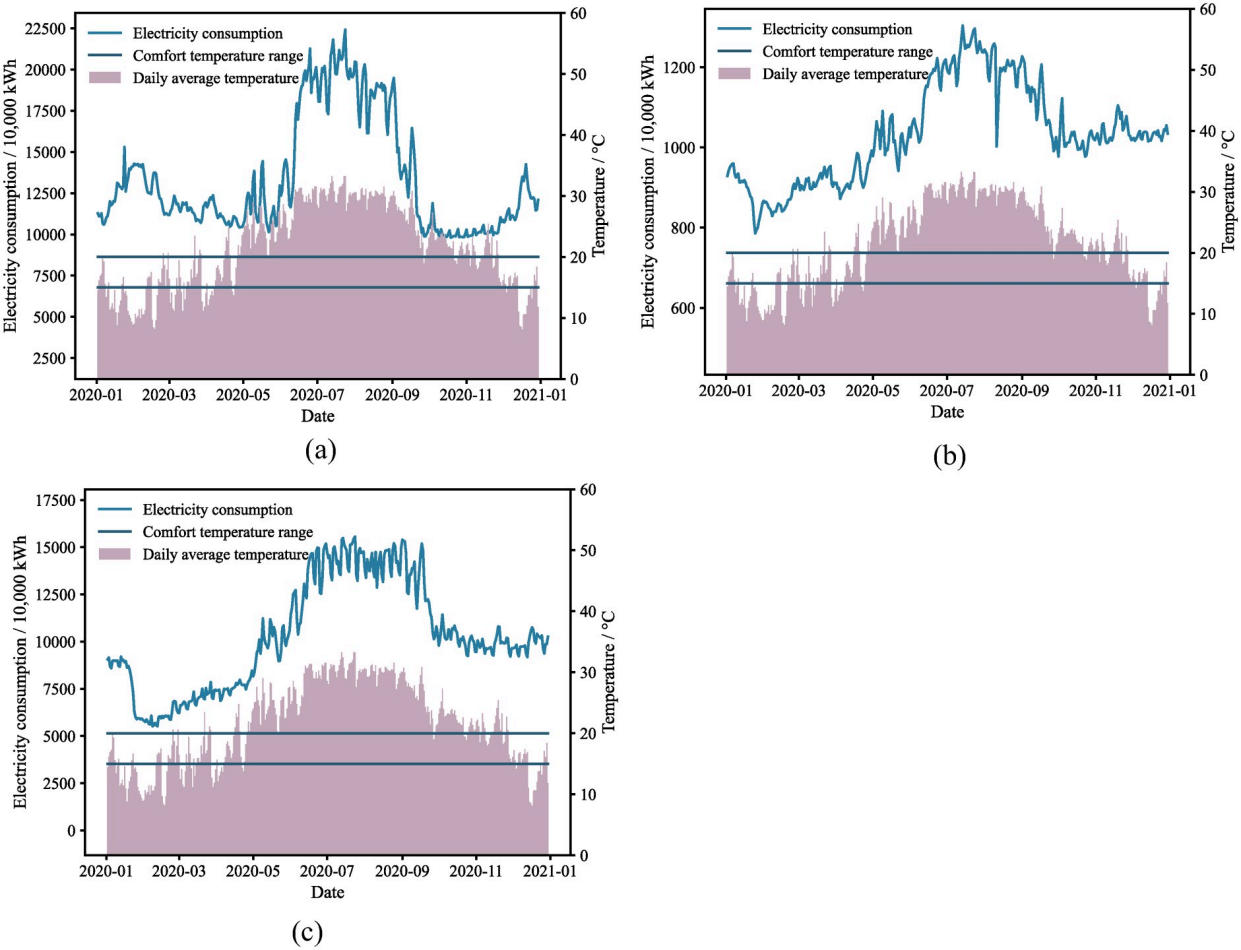
Electricity consumption for different sectors in 2020: (a) Electricity consumption for urban and rural residents; (b) primary sector; (c) tertiary sector.

Fig 2 illustrate the daily average temperature fluctuations and the annual aggregate electricity consumption for urban and rural populations, along with the primary and tertiary sectors in 2020. The figures also annotate the comfort temperature thresholds. These sectors primarily function within the comfort temperature range from March to May and October to November. During these periods, their collective electricity usage serves as the baseline electricity consumption. However, the trends in baseline electricity consumption vary across the sectors: urban and rural residents exhibit relatively stable levels from March to May and October to November, whereas the primary and tertiary sectors typically demonstrate an upward trend. Additionally, there are fluctuations in the average electricity consumption within these sectors from March to May.

In practice, factors such as the rate of increase in the consumer price index (CPI) and year-over-year GDP growth influence baseline electricity consumption [19], leading to complex, nonlinear variations that cannot be precisely captured by a simple linear trend, as assumed by the linear decomposition method. To address this, we employ a polynomial function to model the inherent fluctuations of baseline electricity usage and transform the decomposition of air conditioning electricity consumption into a convex optimization problem.

The linear decomposition method frequently produces negative values for air conditioning electricity consumption when applied to the transitional months of typical days. Since baseline electricity consumption must always be less than or equal to the total grid electricity consumption, we impose an upper limit constraint during the decomposition of air conditioning system power consumption to resolve this issue.

It is noteworthy that the proportion of air conditioning electricity consumption to the total electricity consumption in Wuhan from 2013 to 2018 ranged from 36% to 45%, averaging 40.60% [20]. Given that the share of air conditioning electricity consumption in the total grid electricity consumption is inherently less than 100%, we also implement a lower limit constraint on the baseline electricity consumption.

In summary, it is assumed that the relationship between baseline electricity consumption *C*_*i*_ and time *t*_*i*_ follows a polynomial pattern:

$$\begin{array}{c} C_{i}=\sum_{n=0}^{N}\alpha_{n}t_{i}^{n} \end{array}$$ (3)

where, *α*_*n*_ is a constant. The baseline electricity consumption model is summarized as a constrained convex optimization problem as follows:

$$\begin{array}{c} \left\{ \begin{array}{cc} & \text{min}\;\frac{1}{\left|\phi_{0}\right|}\sum_{i\in \phi_{0}}{\left|\sum_{n=0}^{N}\;\alpha_{n}t_{i}^{n}-E_{i}\right|}^{2}\text{+}\lambda N\\ & s.t.\;pE_{i}\leq \sum_{n=0}^{N}\;\alpha_{n}t_{i}^{n}\leq E_{i},i\in \phi \backslash \phi_{0} \end{array}\right. \end{array}$$ (4)

where, |*ϕ*_0_| is the number of elements in the dataset of typical days; λ is the non-negative weight factor; *p* is the lowest ratio of baseline electricity consumption *C*_*i*_ to total grid electricity consumption. The formula for *p* is as follows:

$$\begin{array}{c} p=1-\frac{\text{max}\left(E_{7},E_{8},E_{9}\right)-E_{3}}{\text{max}(E_{9},E_{8},E_{7})} \end{array}$$ (5)

where, *E*_3_, *E*_7_, *E*_8_ and *E*_9_ are the average total grid electricity consumption for March, July, August, and September, respectively. By deducting the baseline electricity use from the overall electricity consumption, the final electricity consumption for air conditioning is found. This method is referred to as the constrained decomposition method for air conditioning electricity consumption, and abbreviated as constrained decomposition method.

### Apparent temperature and effective duration

Numerous studies [8–11, 20] underscore a robust correlation between air conditioning electricity consumption and ambient temperature. The decision to activate or deactivate air conditioners by residents in practical scenarios is predominantly swayed by external temperature. Consequently, ambient temperature emerges as a pivotal factor influencing air conditioning electricity consumption. However, the majority of air conditioning regulation hinges on individuals’ subjective temperature perception. To more precisely assess how the human body experiences external temperature, the concept of apparent temperature is introduced. Apparent temperature quantifies the sensation of warmth or cold experienced by the human body and was initially proposed by Steadman [21]. Several models for apparent temperature exist in the literature, including the generic heat climate index model [22]. In this study, a segmented apparent temperature model is employed: 1) When the outside temperature is below 50°F, the apparent temperature is dictated by wind speed and the wind chill index (WCI) [23]; 2) For ambient temperatures between 50°F and 80°F, the apparent temperature is equivalent to the ambient temperature; 3) When the ambient temperature exceeds 80°F, the apparent temperature is computed using the heat index (HI) [23]. Apparent temperature can be formulated as follows:

$$\begin{array}{c} T_{A}=\{\begin{array}{cc} & W_{WCL},\;T_{e}<50\\ & T_{e},\;50<T_{\text{e}}<80\\ & H_{HI},\;T_{e}>80 \end{array} \end{array}$$ (6)

where, *T*_*A*_ is apparent temperature, °F; *T*_*e*_ is ambient temperature, °F; *R*_*H*_ is relative humidity; *W*_*s*_ is wind speed, mph. The formulas for calculating the WCL *W*_*WCL*_ and HI *H*_*HI*_ are given as:

$$\begin{array}{c} \begin{array}{cc} & W_{WCL}=35.74+0.6125\times T_{e}-35.75\times W_{s}^{0.16}\\ & \;+0.4275\times T_{e}\times W_{s}^{0.16} \end{array} \end{array}$$ (7)

$$\begin{array}{c} \begin{array}{cc} & H_{HI}=-42.38+2.049\times T_{e}+10.14\times R_{H}\\ & \;-0.2248\times T_{e}\times R_{H}-6.838\times 10^{-3}\times {T_{e}}^{2}\\ & \;-5.482\times 10^{-2}\times R_{H}^{2}\\ & \;+1.228\times 10^{-3}\times {T_{e}}^{2}\times R_{H}\\ & \;+8.528\times 10^{-4}\times T\times R_{H}^{2}\\ & \;-1.99\times 10^{-6}\times {T_{e}}^{2}\times R_{H}^{2} \end{array} \end{array}$$ (8)

The duration for which an air conditioner operates is a significant factor affecting its electricity consumption. The impact of apparent temperature on air conditioning electricity use in practical settings can be summarized as follows: as the apparent temperature increases beyond the comfort range, residents are more likely to switch on their air conditioners, leading to higher electricity consumption. Conversely, when the apparent temperature falls back within the comfort range, residents tend to turn off their air conditioners to conserve electricity. The process can be described as follows: the air conditioner operates continuously and consumes energy when the perceived temperature exceeds the comfort range. This continues until the apparent temperature reenters the desired comfort zone. Based on this, we introduce the concept of the “effective duration of air conditioning,” denoted as $ed_{i}^{m}$, which represents the cumulative time that the apparent temperature remains outside the comfort temperature range.

### Decomposition method for air conditioning electricity consumption based on effective duration

**Fig 3:**
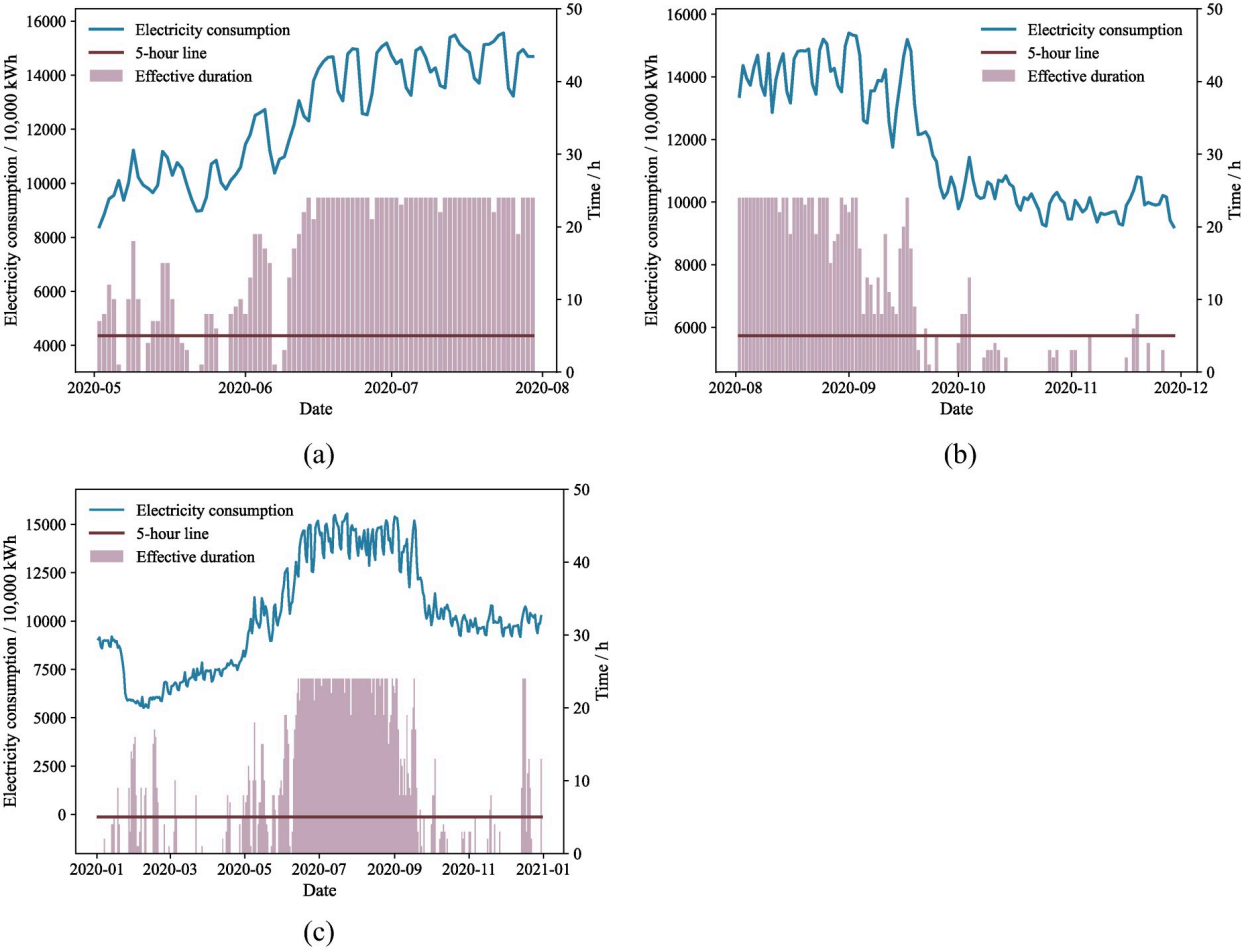
Electricity consumption for tertiary sector in different periods of 2020: (a) spring-summer; (b) summer-autumn;(c) all year.

The variations in the tertiary sector’s electricity consumption and air conditioning effective duration during the spring-summer and summer-autumn transitions in 2020 are illustrated in Fig 3. Fig 3(a) demonstrates that as temperatures and apparent temperature increase during the spring-summer period, the effective duration of air conditioning also rises, leading to higher electricity consumption in the tertiary sector. In Fig 3(b), when the air conditioning effective duration is less than 5 hours, there is a notable decrease in electricity consumption for the tertiary sector. Fig 3(c) shows the relationship between the effective duration of air conditioning and the electricity consumption of the tertiary industry throughout 2020. It is evident in Fig 3(c) that the peak in electricity consumption in the tertiary industry closely aligns with the peak of the air conditioning effective duration. Additionally, fluctuations in the effective duration of air conditioning are mirrored by significant variations in the electricity consumption of the tertiary industry. At the beginning of 2020, the Spring Festival holiday and the COVID-19 pandemic led to many residents staying at home, affecting the tertiary industry. Consequently, there was a marked reduction in the electricity consumption of the tertiary industry, making it challenging to observe the correlation between the effective duration of air conditioning and the electricity consumption of the tertiary industry during this period. In this study, it is assumed that no air conditioning power is used on a day when the time within the comfort temperature range exceeds or equals 19 hours, i.e., when the effective duration of the air conditioning system is less than 5 hours. This criterion is used to define a typical day, addressing seasonal constraints on typical days.

As a result, we include the impact of effective duration in our baseline power consumption model Eq (4) to improve the precision of our air conditioning electricity consumption estimates. In this case, air conditioning electricity consumption can be expressed as a polynomial of effective duration:

$$\begin{array}{c} \left\{ \begin{array}{cc} & C_{i}=\sum_{n=0}^{N}\alpha_{n}t_{i}^{n}\\ & A_{i}=\sum_{m=0}^{M}\beta_{m}ed_{i}^{m} \end{array}\right. \end{array}$$ (9)

where, *β*_*m*_ is a constant; $ed_{i}^{m}$ is the effective duration of air conditioning. Finally, air conditioning electricity consumption is determined by solving the following constrained convex optimization problem:

$$\begin{array}{c} \left\{ \begin{array}{cc} & \text{min}\;(\begin{array}{cc} & \frac{1}{\left|\phi_{0}\right|}\sum_{i\in \phi_{0}}{\left|\sum_{n=0}^{N}\;\alpha_{n}t_{i}^{n}-E_{i}\right|}^{2}\\ & +\frac{1}{\left|\phi \backslash \phi_{0}\right|}\sum_{i\in \phi \backslash \phi_{0}}{\left|\sum_{n=0}^{N}\;\alpha_{n}t_{i}^{n}+\sum_{m=0}^{M}\;\beta_{m}ed_{i}^{m}-E_{i}\right|}^{2}\\ & +\lambda N+\eta M \end{array})\\ & s.t.\;0\leq \sum_{n=0}^{N}\;\alpha_{n}t_{i}^{n}\leq E_{i},\;i\in \phi \backslash \phi_{0} \end{array}\right. \end{array}$$ (10)

where, |*ϕ*∖*ϕ*_0_| is the number of elements in the dataset of atypical days; *η* is a non-negative constant. This method is referred to as the air conditioning electricity consumption decomposition method based on effective duration, and abbreviated as effective duration decomposition method.

## Evaluation index of air conditioning power consumption

Cross-validation cannot be used to verify the efficacy of decomposition techniques for air conditioning power consumption because real data on this topic is not available. Currently, a different method is to use an assessment index created based on pertinent influencing aspects to determine how successful air conditioning electricity consumption decomposition is.

This section introduces an improved consistency index.

### Original consistency index

If (*x*_1_ − *x*_2_)(*y*_1_ − *y*_2_)>0, the observed values (*x*_1_, *y*_1_) and (*x*_2_, *y*_2_) of the random variable (*X*, *Y*) are considered consistent; if (*x*_1_ − *x*_2_)(*y*_1_ − *y*_2_) < 0, the observed values (*x*_1_, *y*_1_) and (*x*_2_, *y*_2_) of the random variable (*X*, *Y*) are inconsistent. Let (*X*_1_, *Y*_1_) and (*X*_2_, *Y*_2_) be independent and identically distributed random variables. Then, Kendall’s *τ* coefficient [24–26] can be defined as:

$$\begin{array}{c} \tau \text{=}P((X_{1}-X_{2})(Y_{1}-Y_{2})>0)-P((X_{1}-X_{2})(Y_{1}-Y_{2})<0) \end{array}$$ (11)

If the bivariate copula [24, 27–29] of (*X*_1_, *Y*_1_) is *C*_1_(*u*, *v*), and that of (*X*_2_, *Y*_2_) is *C*_2_(*u*, *v*), then

$$\begin{array}{c} \tau =4\underset{I^{2}}{\;\int \int \;}C_{2}\left(u,v\right)dC_{1}\left(u,v\right)-1 \end{array}$$ (12)

where, *I* ∈ [0, 1]. Kendall’s *τ* coefficient measures the consistency of the changes of the variable (*X*, *Y*). A larger absolute value of *τ* indicates a stronger consistency between the changes of *X* and *Y*. A positive value of *τ* suggests that the forward changes of *X* and *Y* are consistent, while a negative value of *τ* suggests that the reverse changes of *X* and *Y* are consistent.

### Improved consistency index

The Kendall’s *τ* coefficient mentioned above quantifies the consistency between the variations in air conditioning electricity consumption and the fluctuations in effective duration. However, it does not penalize for erroneous results, such as negative values of air conditioning electricity consumption. Consequently, this paper introduces a revised consistency indicator that incorporates a penalty for negative values of air conditioning electricity consumption.

Based on Kendall’s *τ* coefficient, we incorporate a judgment for negative data values. Let *N* denote the number of samples for the random variable (*X*, *Y*). If all observed values *x*_1_, *x*_2_, *y*_1_ and *y*_2_ are positive and (*x*_1_ − *x*_2_)(*y*_1_ − *y*_2_) > 0, then variables (*x*_1_, *y*_1_) and (*x*_2_, *y*_2_) are a pair of improved consistency variables; if there are negative values in observed values *x*_1_, *x*_2_, *y*_1_ and *y*_2_ or (*x*_1_ − *x*_2_)(*y*_1_ − *y*_2_) < 0, then variables (*x*_1_, *y*_1_) and (*x*_2_, *y*_2_) are a pair of improved inconsistent variables. Under the assumption that longer effective duration of air conditioning operation results in more air conditioning electricity consumption, the improved consistency index *σ* is defined as:

$$\begin{array}{c} \sigma =\frac{N_{\text{c}on}-N_{inc}}{C_{N}^{2}} \end{array}$$ (13)

where, *N*_c*on*_ is the number of improved consistent variable pairs; *N*_*inc*_ is the number of improved inconsistent variable pairs. This index provides a more objective evaluation of the efficacy of the decomposition of air conditioning electricity consumption by reflecting the degree of consistency between air conditioning electricity consumption and the effective duration of air conditioning operation while accounting for the penalty for negative values.

## Experimental results

To verify the efficacy of the suggested approach and index, comparative experiments are conducted in this section using data on power usage in the province of Fujian. The experiments include: 1) Comparative experiment of BLCM, linear decomposition method, constrained decomposition method, and effective duration decomposition method; 2) Comparative experiment of the original consistency index and the improved consistency index; 3) The effects of the original typical day selection rule and the effective duration-based typical day selection rule on the power consumption breakdown findings of air conditioning are compared in this experiment. For the sake of narration, the linear decomposition method, the constrained decomposition method, and the effective duration decomposition method are referred to as Method 1, Method 2, and Method 3, respectively in this section.

### Comparative experiment of air conditioning electricity consumption decomposition methods

The efficacy of the effective duration decomposition method was evaluated in comparison with the BLCM, the linear decomposition method, and the constrained decomposition method. The BLCM designates all dates in March and November as typical days. The linear and constrained decomposition methods select dates in these months with daily average temperatures within the comfort range (15°C-20°C) as typical days. Regardless of the season or month, the effective duration decomposition method identifies typical days as those with air conditioning effective durations of fewer than 5 hours. The experiment utilizes actual electricity consumption data from Fujian Province for the years 2019 to 2020.

**Fig 4:**
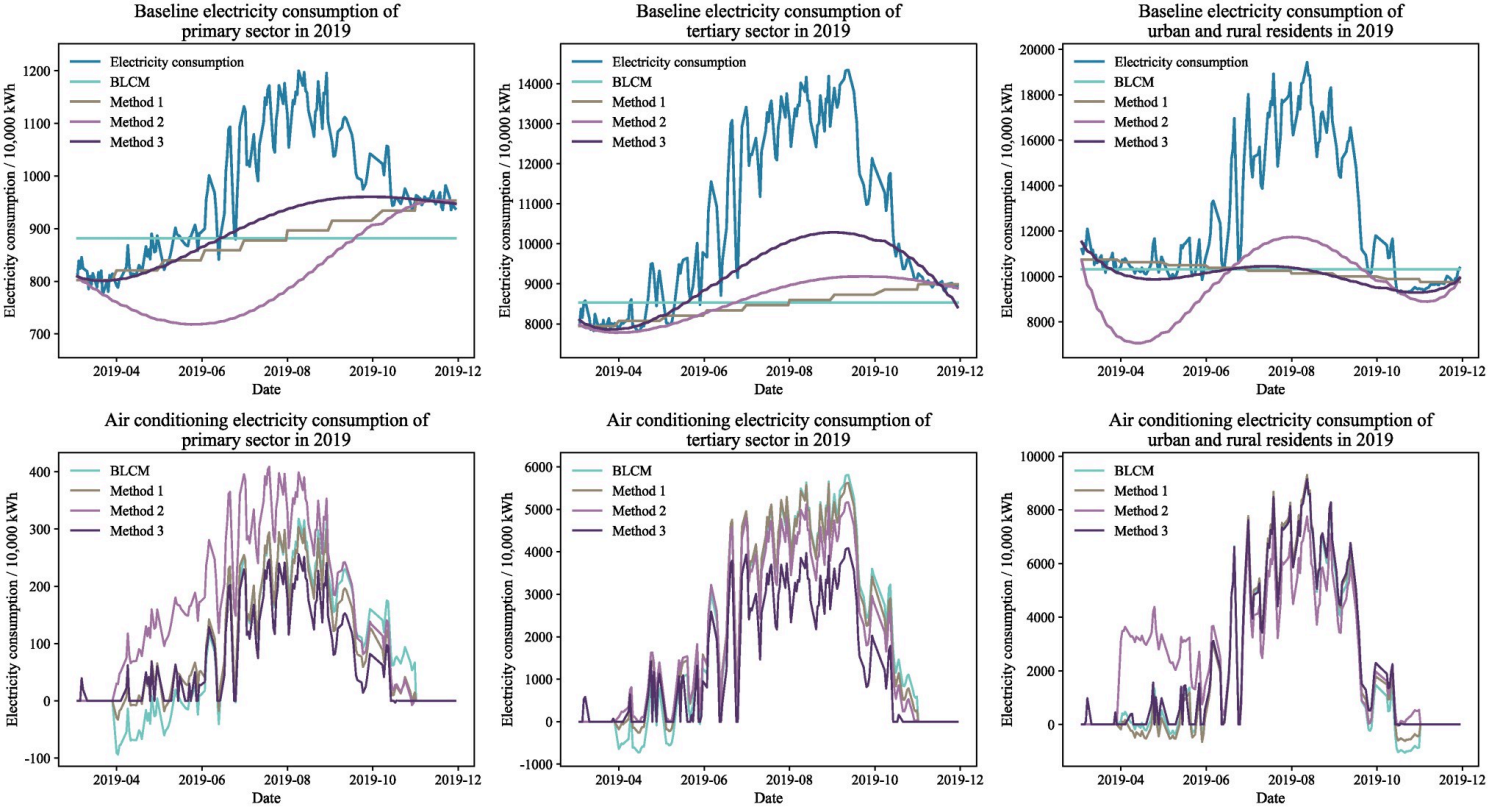
Electricity decomposition results for workdays in 2019.

Figs 4 through 7 display the baseline and air conditioning electricity consumption data across different years and sectors. These results are based on the four methods of decomposing electricity consumption. From the baseline electricity consumption graphs, it is evident that the BLCM’s baseline electricity appears as a straight line, which fails to represent the natural growth in baseline electricity consumption over the calculation period. The linear decomposition method adjusts baseline electricity consumption in a stepwise manner each month, considering the natural growth in electricity consumption. However, the correlation between the fluctuations in baseline electricity consumption and total electricity consumption is relatively low, making the results of the linear decomposition method inconsistent with the actual situation. The baseline electricity consumption curve generated by the constrained decomposition method is smoother; however, the fluctuation trend for specific sectors and years—such as the primary sector in 2019 and the tertiary sector in 2020—slightly diverges from the total electricity consumption. In contrast, the effective duration decomposition method yields a baseline electricity consumption curve that closely aligns with the total electricity consumption trend. This method effectively captures natural changes in baseline electricity consumption, resulting in a smooth and consistent baseline consumption curve.

**Fig 5:**
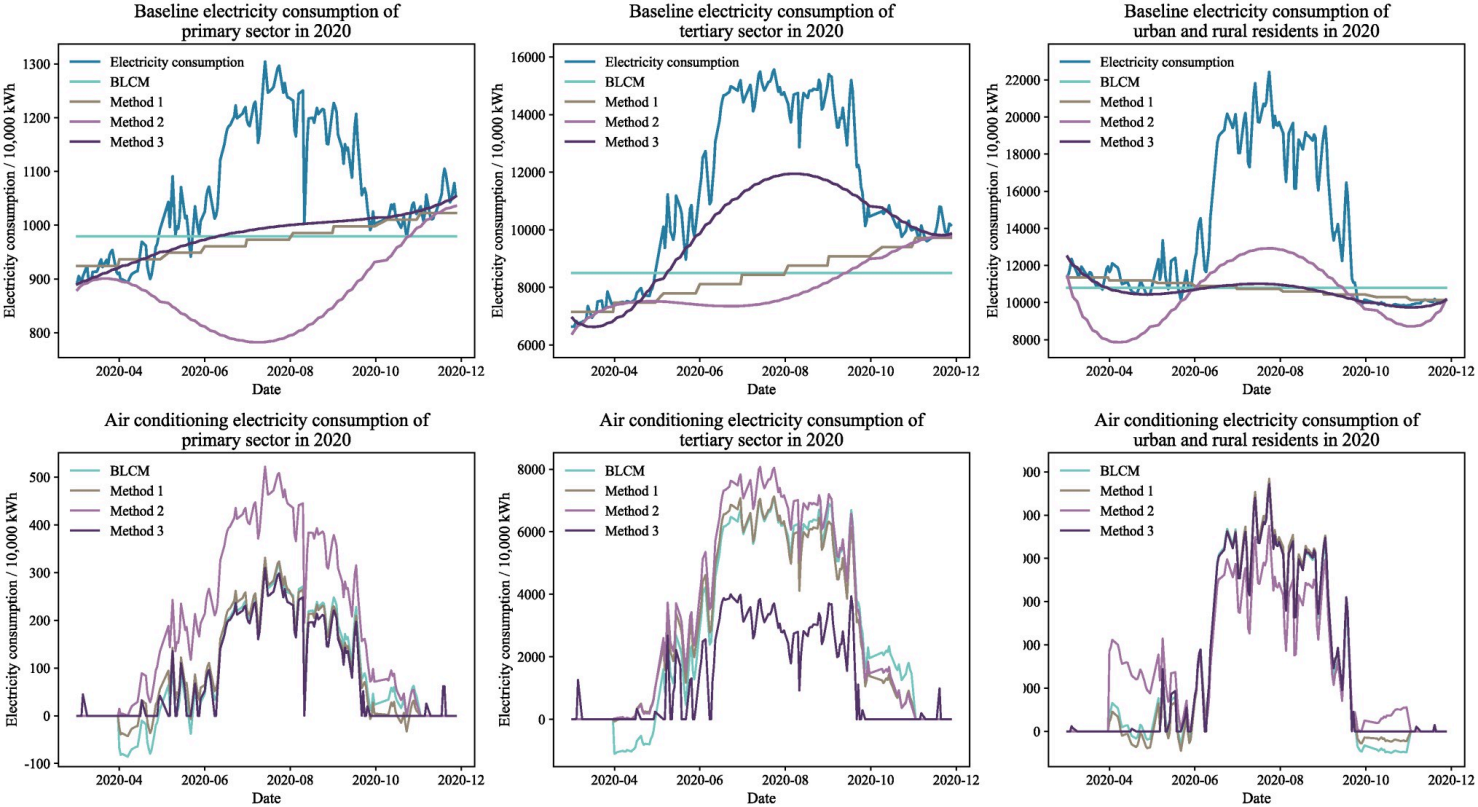
Electricity decomposition results for workdays in 2020.

**Fig 6:**
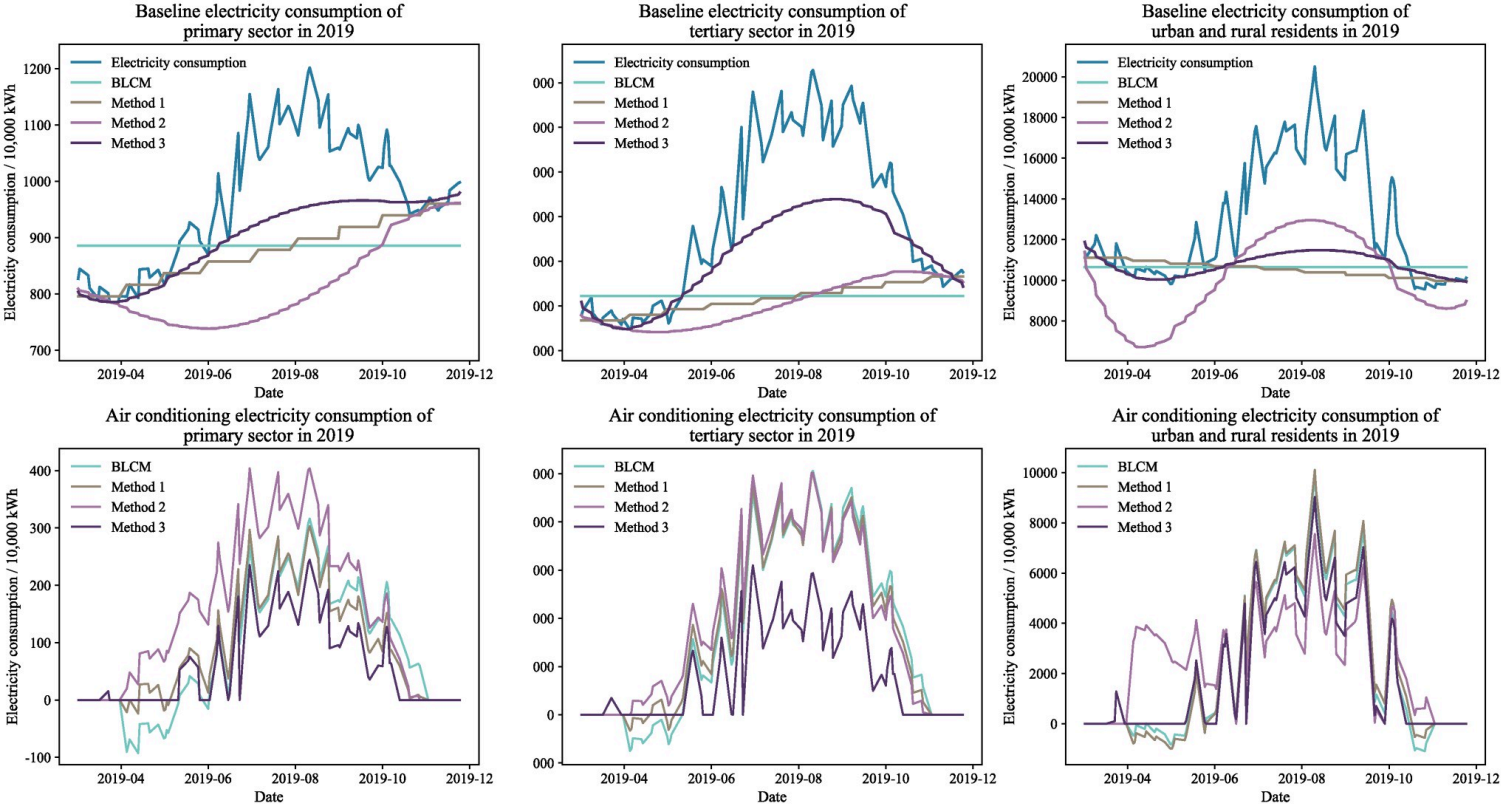
Electricity decomposition results for holidays in 2019.

**Fig 7:**
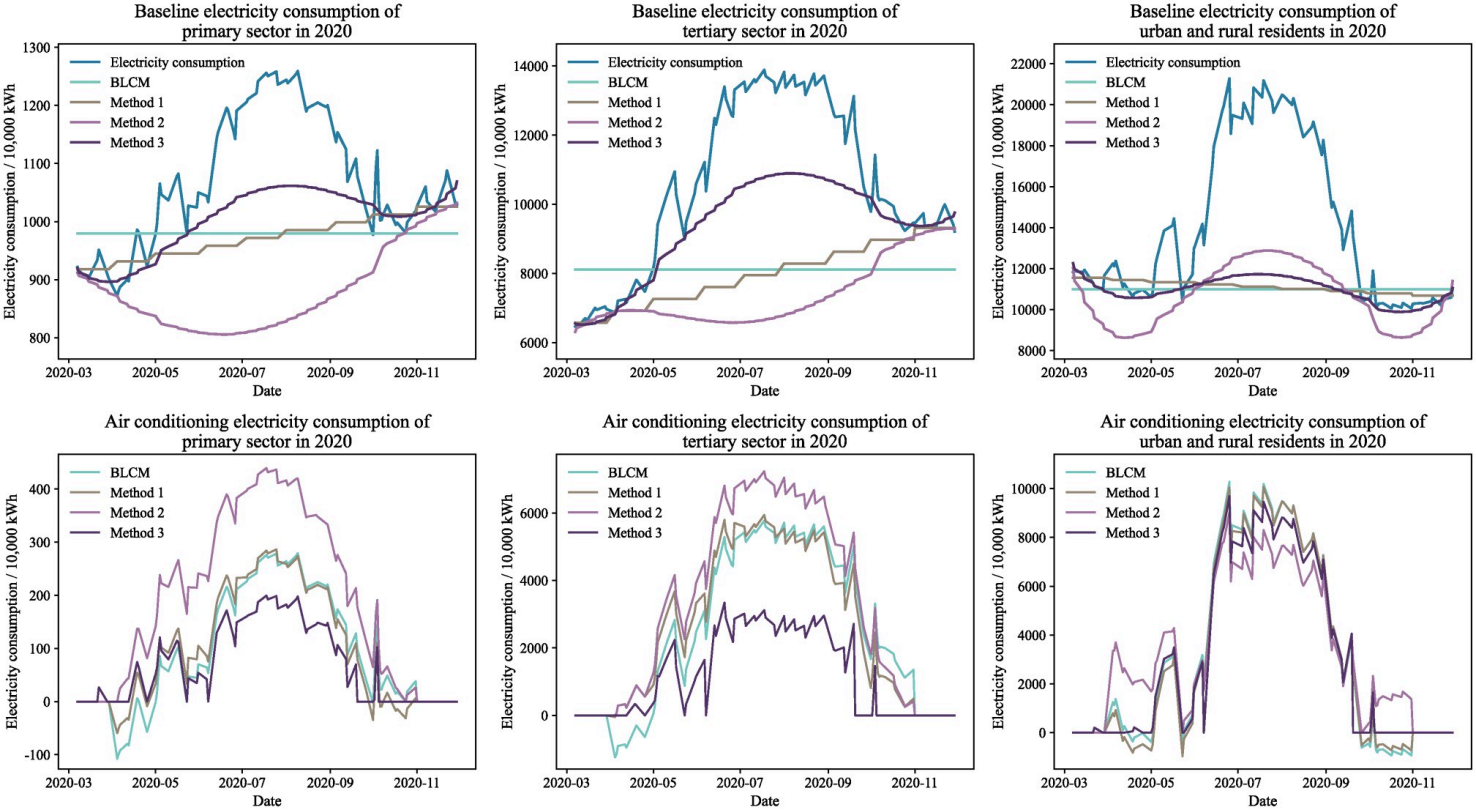
Electricity decomposition results for holidays in 2020.

The effective duration decomposition method yields significantly lower air conditioning electricity consumption statistics compared to the other three methods, based on the calculated results for the primary and tertiary sectors. The primary reason for this discrepancy is the simultaneous operation of numerous high-energy-consuming devices, other than air conditioning, in the actual production processes of these sectors. Consequently, the proportion of power used for air conditioning in the overall electricity consumption of these industries is relatively small, making the conclusions drawn from the effective duration decomposition method more credible.

It is noteworthy that both the BLCM and the linear decomposition method provide figures for April and May that exceed the overall electricity consumption, as evidenced by the baseline electricity consumption results for different years. In other words, these two methods generate negative values in the computed air conditioning electricity consumption, which is clearly unreasonable. In contrast, the constrained decomposition method and the effective duration decomposition method rarely produce negative values in their air conditioning electricity consumption results. Therefore, these two methods decompose air conditioning electricity consumption more rationally.

### Validation of evaluation index

To assess the validity of the proposed consistency index, the original and revised consistency indices were computed for the BLCM, linear decomposition method, constrained decomposition method, and effective duration decomposition method. The dataset encompassed workday data from Fujian Province between 2019 and 2022. The time series for the evaluation indices included the air conditioning system’s effective duration series and electricity consumption series. The comparative results are detailed in Tables 1 and 2, with bold numbers indicating the optimal index values.

**Table 1:** Evaluation indices of the four evaluation methods(Electricity consumption for urban and rural residents).

Time	Original consistency index				Improved consistency index			
	BLCM	Method 1	Method 2	Method 3	BLCM	Method 1	Method 2	Method 3
First half of 2019	**0.84**	**0.84**	0.69	0.83	-0.12	-0.17	0.28	**0.63**
Second half of 2019	0.47	0.51	0.51	**0.67**	-0.32	-0.14	-0.15	**0.28**
First half of 2020	0.86	0.85	0.78	**0.89**	-0.25	-0.35	0.24	**0.51**
Second half of 2020	0.81	0.82	**0.83**	0.70	0.01	0.02	-0.01	**0.26**
First half of 2021	**0.84**	0.83	-0.11	0.76	-0.19	-0.19	-0.65	**0.50**
Second half of 2021	**0.68**	0.67	0.66	0.58	-0.08	0.02	0.01	**0.31**
First half of 2022	0.88	0.87	0.77	**0.90**	-0.28	-0.38	0.22	**0.59**
Second half of 2022	0.64	0.62	**0.67**	0.55	0.02	0.01	0.03	**0.40**

**Table 2:** Electricity consumption for urban and rural residents(Tertiary sector).

Time	Original consistency index				Improved consistency index			
	BLCM	Method 1	Method 2	Method 3	BLCM	Method 1	Method 2	Method 3
First half of 2019	0.81	0.82	**0.86**	0.79	0.08	0.18	0.39	**0.62**
Second half of 2019	**0.42**	0.33	0.13	-0.07	-0.62	-0.68	-0.35	**-0.34**
First half of 2020	0.84	0.88	**0.89**	0.81	-0.02	0.27	0.31	**0.47**
Second half of 2020	**0.72**	**0.72**	0.69	0.61	-0.75	-0.66	-0.05	**0.28**
First half of 2021	**0.85**	**0.85**	0.84	0.70	0.24	0.36	0.35	**0.49**
Second half of 2021	0.53	**0.61**	0.46	0.49	-0.23	-0.18	-0.11	**0.21**
First half of 2022	0.86	0.87	**0.88**	0.74	0.05	0.18	0.30	**0.43**
Second half of 2022	0.27	0.28	0.37	**0.45**	-0.91	-0.83	-0.13	**0.17**

Under the original consistency index, the BLCM excels in six items, the linear decomposition method in four, the constrained decomposition method in five, and the effective duration decomposition method in four of the sixteen sub-items across the two sectors. This mixed effectiveness of the decomposition methods for air conditioning electricity consumption complicates the determination of the best method. However, the outcomes of the comparative experiments in the previous section demonstrate that the effective duration decomposition method is superior, suggesting that the original consistency index may not accurately reflect the quality of the air conditioning electricity consumption decomposition methods. In contrast, under the improved consistency index, the effective duration decomposition method leads in the majority of sub-items. This is consistent with the findings from the previous section, which indicate that the enhanced index confirms the superiority of the effective duration decomposition method over the other three methods. Consequently, the enhanced consistency index may provide a more rational and efficient evaluation of the quality of air conditioning electricity consumption decomposition techniques.

### Impact of typical day selection rules on air conditioning electricity consumption

To evaluate the efficacy of the effective duration-based typical day selection rules, this section conducts experiments comparing the original typical day selection rules with the effective duration-based rules for selecting typical days for the effective duration decomposition method. The dataset employed consists of workday electricity consumption data for the tertiary sector and urban and rural residents in 2020.

**Fig 8:**
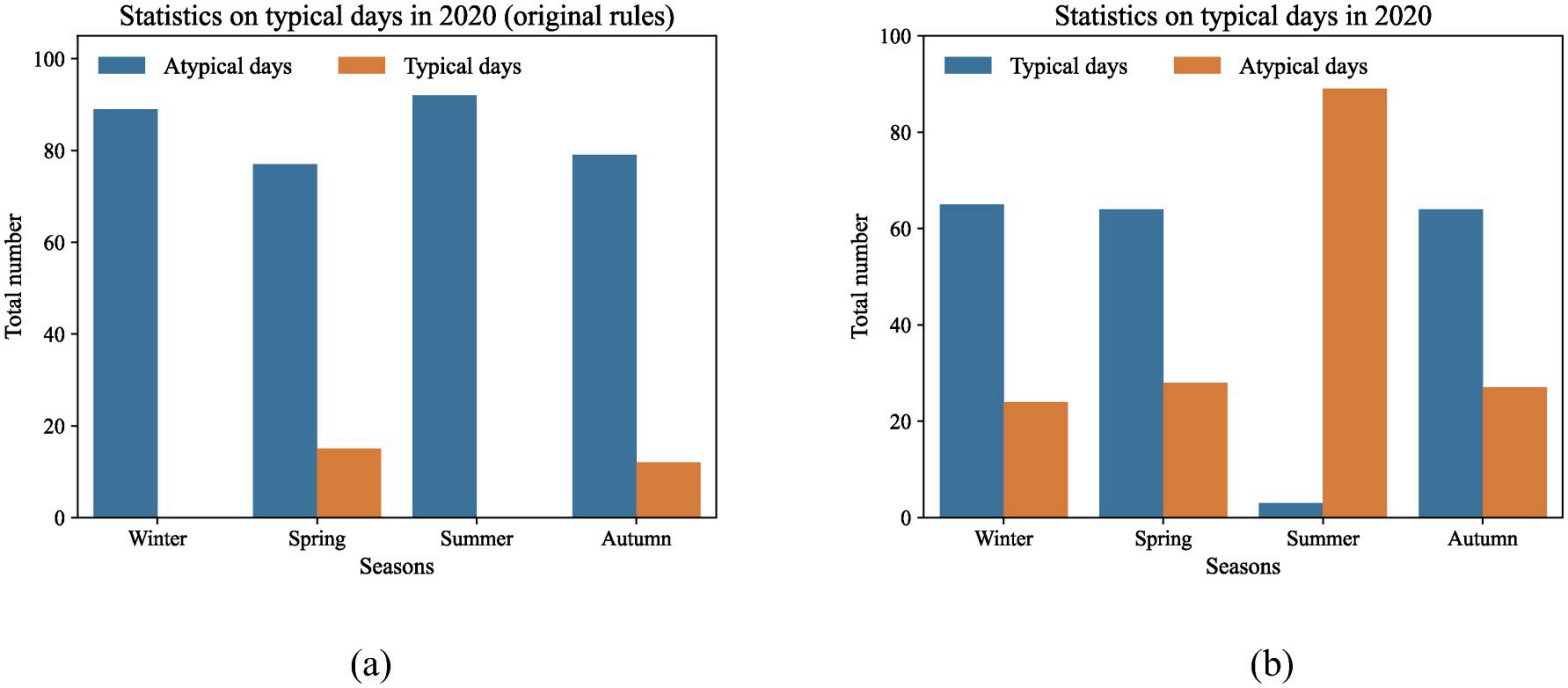
Statistical map of the typical days selected by different rules: (a) original rules; (b) effective duration-based rules.

The statistical distribution of the number of typical days under the two different selection rules is illustrated in Fig 8. As depicted in Fig 8(a), under the original typical day selection rules, typical days are predominantly chosen during the spring and autumn seasons, with a limited number of selections. This scarcity of typical days hampers the provision of a robust dataset for accurately calculating air conditioning electricity consumption. In contrast, Fig 8(b) shows that when using the typical day selection criteria based on effective duration, the data distribution is more evenly spread across the time axis, with typical days selected throughout all seasons. This approach leverages the effective duration of air conditioning based on apparent temperature, resulting in a denser data distribution and enabling the provision of more comprehensive constraint information for the calculation of baseline electricity consumption.

**Fig 9:**
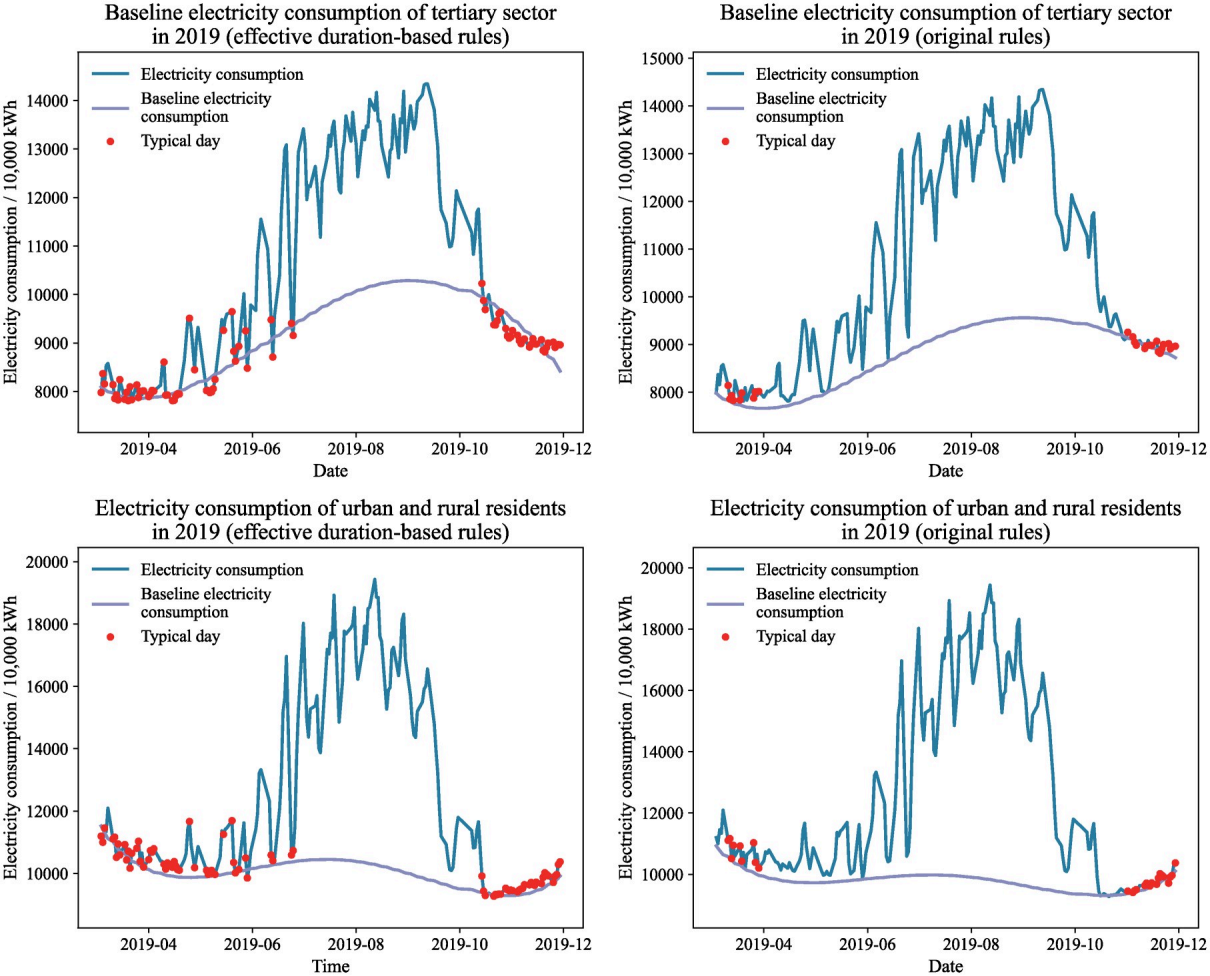
Baseline electricity consumption of 2019 under different typical day selection rules.

**Fig 10:**
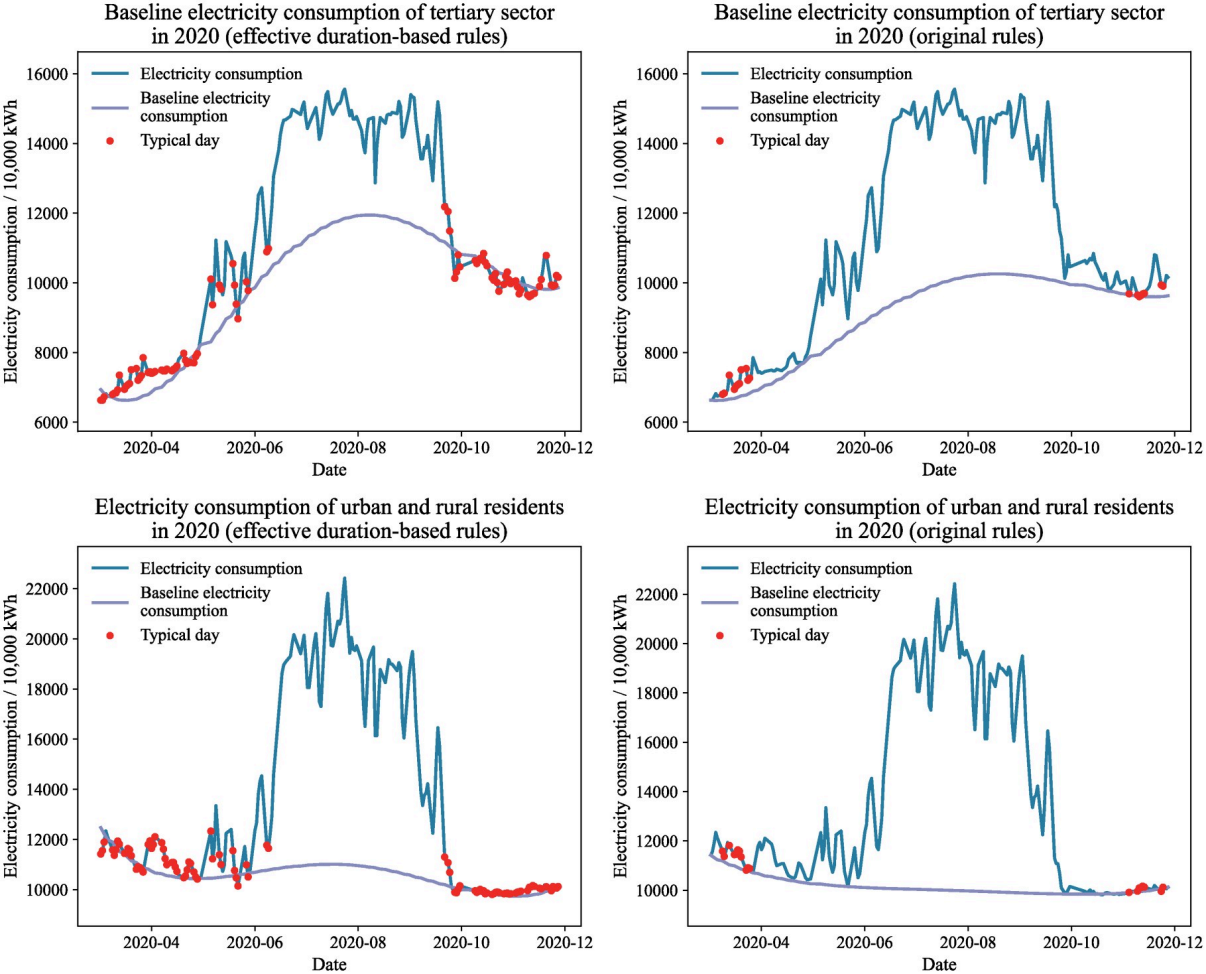
Baseline electricity consumption of 2020 under different typical day selection rules.

Figs 9 and 10 present the baseline power consumption estimates for the tertiary sector and urban and rural inhabitants in 2019 and 2020, respectively, using various average day selection rules. Under the original typical day selection guidelines, the baseline electricity consumption calculated by the effective duration decomposition method shows a fluctuating trend that weakly correlates with the overall power consumption. This is particularly noticeable in the baseline electricity consumption trend for urban and rural residents in 2020, which closely resembles a straight line and does not accurately reflect the actual fluctuations in electricity consumption. In contrast, when using the effective duration-based typical day selection guidelines, the effective duration decomposition method generates a smoother baseline electricity consumption curve that closely follows the total grid electricity consumption changes, better representing the natural variations in baseline electricity.

Upon analyzing the total electricity consumption curves for the tertiary industry and urban and rural residents, it becomes apparent that there are multiple instances of sudden reductions in electricity usage across both sectors. Some of these anomalies are attributable to periods of environmental comfort, during which air conditioning units operate less frequently or are inactive. This leads to a decrease or cessation of air conditioning electricity consumption, which is temperature-dependent, resulting in a sudden drop in total grid electricity consumption. Under the original typical day selection rules, these anomalies are not recognized as typical and impede the accurate calculation of baseline electricity consumption. Conversely, the effective duration-based rules incorporate these anomalies as typical days, providing essential constraint information for the calculation of baseline electricity consumption. Thus, the effective duration-based rules more accurately reflect the actual conditions for the selection of typical days.

The labels of each typical day in Figs 9 and 10 indicate that the months of April through June, along with October and November, have a significantly higher distribution of typical days under the effective duration-based typical day selection rules. Additionally, there are some typical days in July. These typical days provide more constrained information to describe the fluctuation trends of baseline electricity consumption. In contrast, under the original typical day selection rules, typical days are limited and primarily concentrated in March and November, providing insufficient constraint information for midsummer baseline electricity consumption computation, making it difficult to accurately depict the baseline electricity consumption fluctuation trends. Consequently, under the typical day selection rules based on effective duration, the results of air conditioning electricity consumption decomposition are more reasonable.

## Conclusions

This research examines the methodologies utilized for decomposing the electricity consumption associated with air conditioning and evaluates the results of these decomposition processes. An enhancement to the consistency index has been proposed, alongside a decomposition strategy for air conditioning electricity usage that is based on effective duration and apparent temperature. In instances where historical data on air conditioning electricity consumption is unavailable, the proposed method serves to decompose this consumption by leveraging total electricity usage data. Experimental outcomes indicate that, relative to other methods, the proposed approach demonstrates superior performance in analyzing actual electricity consumption data for air conditioning systems. The enhanced consistency index facilitates a more rigorous and efficient assessment of the efficacy of air conditioning electricity consumption decomposition techniques.

The absence of real data for the air conditioning electricity consumption analyzed in this study precludes the optimization of consumption methods through the integration of real electricity data characteristics, as well as the validation of decomposition methods’ effectiveness with real data. Given that electricity consumption is inherently tied to societal operations, policy alterations, extreme weather conditions, and other irregular events can uniquely influence electricity consumption data. These irregular influences are reflected as outliers in the electricity consumption curve. In practical applications, the presence of outliers presents a challenge to the decomposition methods for air conditioning electricity consumption. Future research will focus on the impact of irregular events on air conditioning electricity consumption.

## Supporting information

S1 Data (XLSX)
